# Comprehensive analysis of key host gene-microbe networks in the cecum tissues of the obese rabbits induced by a high-fat diet

**DOI:** 10.3389/fcimb.2024.1407051

**Published:** 2024-06-14

**Authors:** Yanhong Li, Xiaolan Qi, Qinrong Wang, Yan He, Zhupeng Li, Xi Cen, Limin Wei

**Affiliations:** ^1^ Key Laboratory of Endemic and Ethnic Diseases, Ministry of Education & Key Laboratory of Medical Molecular Biology of Guizhou Province, Collaborative Innovation Center for Prevention and Control of Endemic and Ethnic Regional Diseases Co-constructed by the Province and Ministry, Guizhou Medical University, Guiyang, Guizhou, China; ^2^ Chongqing Key Laboratory of High Active Traditional Chinese Drug Delivery System, Chongqing Medical and Pharmaceutical College, Chongqing, China; ^3^ College of Pharmacy, Chongqing Medical University, Chongqing, China

**Keywords:** lipid metabolism, cecum microorganism, fattening model of rabbit, obesity, metagenome

## Abstract

The Cecum is a key site for cellulose digestion in nutrient metabolism of intestine, but its mechanisms of microbial and gene interactions has not been fully elucidated during pathogenesis of obesity. Therefore, the cecum tissues of the New Zealand rabbits and their contents between the high-fat diet-induced group (Ob) and control group (Co) were collected and analyzed using multi-omics. The metagenomic analysis indicated that the relative abundances of *Corallococcus_sp._CAG:1435* and *Flavobacteriales bacterium* species were significantly lower, while those of *Akkermansia glycaniphila*, *Clostridium_sp._CAG:793*, *Mycoplasma_sp._CAG:776*, *Mycoplasma_sp._CAG:472*, *Clostridium_sp._CAG:609*, *Akkermansia_sp._KLE1605*, *Clostridium_sp._CAG:508*, and *Firmicutes_bacterium_CAG:460* species were significantly higher in the Ob as compared to those in Co. Transcriptomic sequencing results showed that the differentially upregulated genes were mainly enriched in pathways, including calcium signaling pathway, PI3K-Akt signaling pathway, and Wnt signaling pathway, while the differentially downregulated genes were mainly enriched in pathways of NF-kappaB signaling pathway and T cell receptor signaling pathway. The comparative analysis of metabolites showed that the glycine, serine, and threonine metabolism and cysteine and methionine metabolism were the important metabolic pathways between the two groups. The combined analysis showed that *CAMK1*, *IGFBP6*, and *IGFBP4* genes were highly correlated with *Clostridium_sp._CAG:793*, and *Akkermansia_glycaniphila* species. Thus, the preliminary study elucidated the microbial and gene interactions in cecum of obese rabbit and provided a basis for further studies in intestinal intervention for human obesity.

## 1 Introduction

Obesity is a global epidemic disease, which is considered a potential threat to the health of middle-aged and elderly people in the future. Gut microbiota is associated with the development of obesity and obesity-related metabolic syndromes, such as NAFLD, insulin resistance, T2DM, hypertension, cardiovascular disease, and coronary heart disease. Numerous factors, such as nutrition diet, genetic expression pattern, age, and biological factors in the body, can affect gut microbiota (Milani et al., 2017; Cuevas-Sierra et al., 2019). However, the changes in dietary fiber can alter the composition of gut microbiota, thereby causing the occurrence of metabolic diseases in the host. Especially, the changes in the gut microbiota in the large intestine are highly related to the fermentation of dietary fibers for the body’s nutrient metabolism and absorption. This fermentation of dietary fibers produces numerous beneficial metabolites, such as SCFAs and succinate, which can be used to intervene in obesity in human (Canfora et al., 2019). Biologically, the production of gut microbial metabolites is closely related to host system evolution and dietary behavior (Yao et al., 2021; Cuomo et al., 2022). Previous studies have confirmed that the plant fibrotic diet is highly associated with the gut microbiota, such as *Roseburia*, *Eubacterium rectale*, and *Faecalibacterium prausnitzii*, which increase the total concentration of SCFAs (David et al., 2014; Wu et al., 2017). Previous study indicated that the *HDACDEL* gene could maintain intestinal homeostasis in mice while interacting with gut microbiota (Wu et al., 2020). The cecum tissues also play numerous functional roles in intestinal nutrient absorption; however, the interactions between gut microbiota and host genes are not fully understood yet.

In obese populations, excessive lipid deposition in the body of the patients is a major factor inducing metabolic diseases. The long-term supplementation of high-fat and high-sugar diets increases the metabolic burden on various organs in the body, especially leading to the ectopic deposition of visceral fat, which causes the occurrence of metabolic diseases (Neeland et al., 2019). Clinical diagnosis also shows that excessive fat deposition in the intestine can affect intestinal peristalsis, disrupt intestinal homeostasis, and damage the intestinal mucosal barrier (Festi et al., 2020; Chen et al., 2021). Studies have shown that a large amount of lipids accumulate in the intestine of obese patients, and these adipose tissues can secrete pro-inflammatory factors, such as *TNF-α* and *IL-6*, and anti-inflammatory factor (*Adiponectin*). These pro- and anti-inflammatory factors can affect the intestinal environment and produce different types of secondary metabolites to rebalance the nutritional metabolism of gut microbiota (Bisanz et al., 2019; Liu et al., 2022; Bodilly et al., 2023). Simultaneously, the different gut microbial metabolites are a major factor regulating the homeostasis of the intestinal environment. Previous studies indicated that the specific knockout of *HIF-2α* in mice could disturb the intestinal lactic acid level, thereby decreasing the abundance of Bacteroides and increasing that of rumen cocci, which resulted in promoting the levels of taurocholic acid and deoxycholic acid (Wu et al., 2021). These acids could activate the adipose G-protein-coupled bile acid receptor and upregulate the expression of *UCP1* and *CKMT2*, increasing heat production in white adipose tissue (Haddish and Yun, 2023). *Clostridium butyricum CCFM1299* could reduce obesity by increasing energy expenditure and regulating host bile acid metabolism (Liao et al., 2023). Simultaneously, studies confirmed that bile acids, butyric acid, succinate, cinnabarinic acid, urolithin A, and asparagine produced by gut microbiota were involved in regulating metabolic pathways in adipose tissue cells, such as PKA, PPAR-alpha, PGC-1α, and mTORC1 signaling pathways (Thibaut and Bindels, 2022; Tang et al., 2023). However, the circulatory metabolic system and cooperative mechanism among gut microbiota, gut metabolites, and host genes in the pathogenesis of diet-induced obesity have not been fully understood yet.

As an important part of the large intestine, the cecum tissues play an important role in the digestion and reabsorption of food nutrition. Studies have shown that the cecum of rabbits is well-developed and can be used as a good intestinal model to study the pathogenesis of animal digestive diseases (Dabbou et al., 2019; Abdel-Kafy et al., 2023). Therefore, this study aimed to find the interaction mechanisms among host genes and gut microbiota in the cecum tissues of rabbits fed with a high-fat diet in order to provide a basis for improving and intervening in animal and human intestinal metabolic diseases.

## 2 Materials and methods

### 2.1 Construction of the rabbit model

Experiment design. A total of 22 male New Zealand rabbits aged 270 days were randomly selected and divided into two groups: a control group (Co, n = 11) and a high-fat group (Ob, n = 11). All the rabbits were fed in the animal experiment center of Guizhou Medical University, reared in the same breeding management conditions (The optimal indoor feeding conditions: temperature at 15 ~ 25°C, humidity at 60% ~ 70% and 12 hours a day alternating light), and vaccinated regularly. The experiment was divided into pre-feeding (14 days) and experimental (28 days) periods.

**Figure 1:**
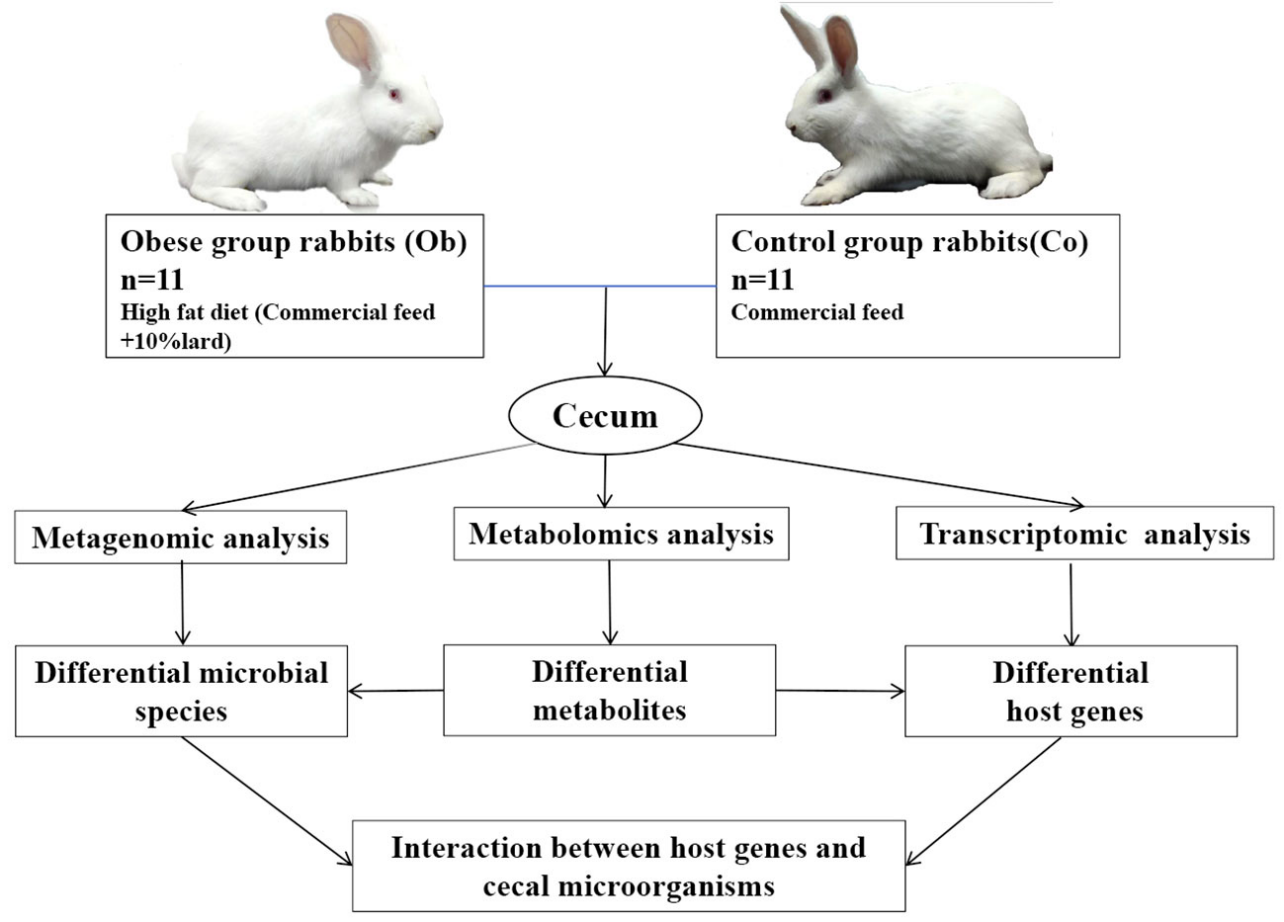
Design route of fattening experiment with induced by a high fat diet in adult New Zealand rabbits.

Feeding protocol. The experiment was divided into a 14-day pre-feeding period and a 28-day experimental period. The rabbits were separately housed in single cages and fed (200 g per rabbit) twice a day. During the pre-feeding period, all the rabbits were fed 200 g of commercial diet twice a day per rabbit, and the specific feed composition is provided in **Table 1**. During the experimental period, the Ob rabbits were fed with a high-fat diet (Commercial feed + 10% lard), while the Co rabbits were fed with the commercial feed only. The feeding amount was recorded until the end of the experimental period. The specific experiment design was shown in the **Figure 1**.

**Table 1:** Commercial feed nutrient composition ratio.

Ingredients	H_2_O	Crude protein≥(%)	Crude fibre	Crude ash	Ca%	Total phosphorus	NaCl%	Lysine	Cystine and methionine(≥%)
	(≤%)		(≤%)	(≤%)		(≥%)		(≥%)	
Content	14	14	7.0-20.0	14	0.6-1.50	0.4	0.3-0.80	0.6	0.5

### 2.2 Total RNA extraction and RNA-sequencing

Total RNA was extracted from 6 cecum samples obtained from Co and Ob groups using the TRIZOL reagent (Invitrogen, CA, USA) following the manufacturer’s instructions. The quantification and assessment of RNA purity were performed using the NanoDrop 2000 spectrophotometer (Thermo Scientific, USA). Then, the libraries were constructed using VAHTS Universal V6 RNA-seq Library Prep Kit following the manufacturer’s instructions. The RNA sequencing was performed by OE Biotech Co., Ltd. (Shanghai, China). The libraries were sequenced on an Illumina NovaSeq 6000 platform, generating 150-bp paired-end reads. The raw sequence reads in FastQ format were first processed using fastp, and the low-quality reads were filtered out to acquire a clean read for each sample. The clean reads were mapped to the reference genome (Oryctolagus cuniculus UM_NZW_1.0) using HISAT2 software. FPKM of each gene was calculated, and the read counts of each gene were obtained using HTSeq-count.

### 2.3 DNA extraction and metagenomic sequencing

Total DNA was extracted from 12 cecal content samples obtained from Co and Ob groups using a QIAamp® Fast DNA Stool Mini Kit (Qiagen, Hilden, Germany) following the manufacturer’s instructions. The DNA concentration and integrity were assessed using the NanoDrop2000 spectrophotometer (Thermo Fisher Scientific, Waltham, MA, USA) and agarose gel electrophoresis, respectively. DNA was fragmented using an S220 Focused-ultrasonicator (Covaris, USA) and cleaned up using Agencourt AMPure XP beads (Beckman Coulter Co., USA). The libraries were prepared using TruSeq Nano DNA LT Sample Preparation Kit (Illumina, San Diego, CA, USA) following the manufacturer’s instructions and sequenced on an Illumina NovaSeq 6000 platform. The metagenome sequencing was performed by OE Biotech Co., Ltd. (Shanghai, China).

### 2.4 Metabolite extraction and liquid chromatography mass spectrometry analysis

A total of 60 mg of sample was weighed from each of the 12 samples obtained from the Co and Ob groups and placed into a 1.5-mL EP tube. The precooled 600 μL and 300 μL methanol-water (v/v, 4:1, containing 4 μg/mL L-2-chlorophenylphenylalanine) were used for cold treatment, centrifugation, and filtration, respectively, and the samples were then prepared for LC-MS. Quality control (QC) samples were prepared by mixing the extracts of all samples in equal volumes. The metabolites were detected using an LC-MS system composed of ACQUITY UPLC I-Class plus ultra-high performance liquid series QE and high-resolution mass spectrometer. The ACQUITY UPLC HSS T3 column (100 mm × 2.1 mm, 1.8 µm) was used with a column temperature of 45°C chromatography. A 5-μL sample was injected at a flow rate of 0.35 mL/min, and the detection was performed at different time points through different liquid phase gradients (mobile phase A was water (containing 0.1% formic acid), and mobile phase B was acetonitrile). ESI was used as an ion source to detect the sample quality spectrum signal, and the positive and negative ion scanning modes were used.

### 2.5 HE staining

The six cecal tissue samples from Co and Ob group were fixed with 4% paraformaldehyde solution and rinsed with running water for tissue trimming. Then, placed them in a pathological plastic box for embedding to dehydrate (75% ethanol for 6 h, 85% ethanol for 10 h, 95% ethanol for 4 h, absolute ethanol I for 2 h, absolute ethanol II for 2 h), transparent (xylene I for 20 min, xylene II for 15 min), immerse in wax for 3 h, and make them into tissue wax blocks. The tissue wax blocks were cut into 5 µm thick slices using a Leica RM2235 microtome, fixed on a glass slide (at least 2 hours at 60°C). The unstained sections were deparaffinized with xylene, washed with running water for 20 min, stained with hematoxylin for 30 min, washed with running water for 20 min, differentiated with hydrochloric acid alcohol, stained with eosin for 5 min, finally dehydrated with gradient alcohol, transparent with xylene and sealed with resin glue. The stained sections were photographed and recorded using a fluorescence inverted microscope.

### 2.6 Bioinformatic analysis

#### 2.6.1 Analysis of RNA-sequencing data

The genes in RNA-seq data were filtered using NOISeq and DESeq2 software. The filtered genes were further selected based on the threshold values (∣log2FC∣≥1 and FDR <0.05). The gene enrichment analysis was performed using DAVID (https://david.ncifcrf.gov/tools.jsp) and the KEGG database (Kyoto Encyclopedia of Genes and Genomes). The R software package was used to draw diagrams.

#### 2.6.2 Analysis of metagenomic data

All the original sequence data obtained from the libraries were trimmed and filtered using Trimmomatic (v 0.36) (Bolger et al., 2014). The post-filtered pair-end clean reads were aligned against the reference genome (Oryctolagus cuniculus UM_NZW_1.0) using bowtie2 (v2.2.9), and the aligned reads were discarded. Metagenome assembly was constructed using MEGAHIT (v1.1.2) (Li et al., 2015), and the new Scaftigs with >500 bp length were screened after getting valid reads. The ORF prediction of assembled scaffolds was performed using prodigal (v2.6.3) (Hyatt et al., 2010). CDHIT (v 4.5.7) was used to predict genes from the non-redundant gene sets. The taxonomy of the species was obtained by searching in the NR Library, and its abundance was calculated using the corresponding abundance of the genes. Then, the abundance of microbiota was identified at the phylum, class, order, family, genus, and species levels. The PCA analysis and plotting of the taxonomy abundance spectrum or functional abundance spectrum were performed using R software (v 3.2.0). The linear discriminant analysis effect size (LEfSe) method was used to compare the taxonomy abundance spectrum or functional abundance spectrum.

#### 2.6.3 Metabolite function analysis

The unique metabolites in the original LC-MS data were processed and identified using Progenesis QI software v2.3 (Nonlinear, Dynamics, Newcastle, UK). The main parameters included 5 ppm precursor tolerance, 10 ppm product tolerance, and 5% product ion threshold. The compounds were identified based on the precise mass-to-charge ratio (M/z), secondary fragments, and isotopic distribution using The Human Metabolome Database (HMDB), Lipidmaps (V2.3), Metlin, EMDB, and PMDB for qualitative analysis. The compounds with scores below 36 (out of 60) points were deemed inaccurate and removed. Orthogonal Partial Least-Squares-Discriminant Analysis (OPLS-DA) and Partial Least-Squares-Discriminant Analysis (PLS-DA) were used to identify the differential metabolites between the groups. Variable Importance of Projection (VIP) values obtained from the OPLS-DA model were used to rank the overall contribution of each variable to group discrimination. A two-tailed Student’s t-test was used to verify the significant differences in the metabolites between the groups. The differential metabolites were selected based on VIP values >1.0 and P-values <0.05.

### 2.7 Correlation network construction

The protein-protein interaction network obtained using STRING (https://string-db.org/cgi/input.pl) website and Cytoscape software was screened for the highly related genes using Cytohubba. In order to search the key genes in a network, Cytohubba uses 12 algorithms, including degree, edge percolated component (EPC), maximum neighborhood component (MNC), density of maximum neighborhood component (DMNC), maximal clique centrality (MCC), and centralities based on shortest paths, such as bottleneck (BN), eccentricity, closeness, radiality, betweenness, and stress.

### 2.8 Statistical analysis

The SPASS 22.0 software was used to analyze the significance of differences between the groups using student’s t-test. All statistics were based on mean ± standard error (Mean ± SE). *P* < 0.05 and *P* < 0.01 were considered as significant difference “*” and extremely significant “**”, respectively. The results were mapped using GraphPad Prism8 software.

## 3 Results

### 3.1 Comparative analysis of body weight, visceral fat deposition and cecal pathological diagnosis among the conditions of rabbits

**Figure 2:**
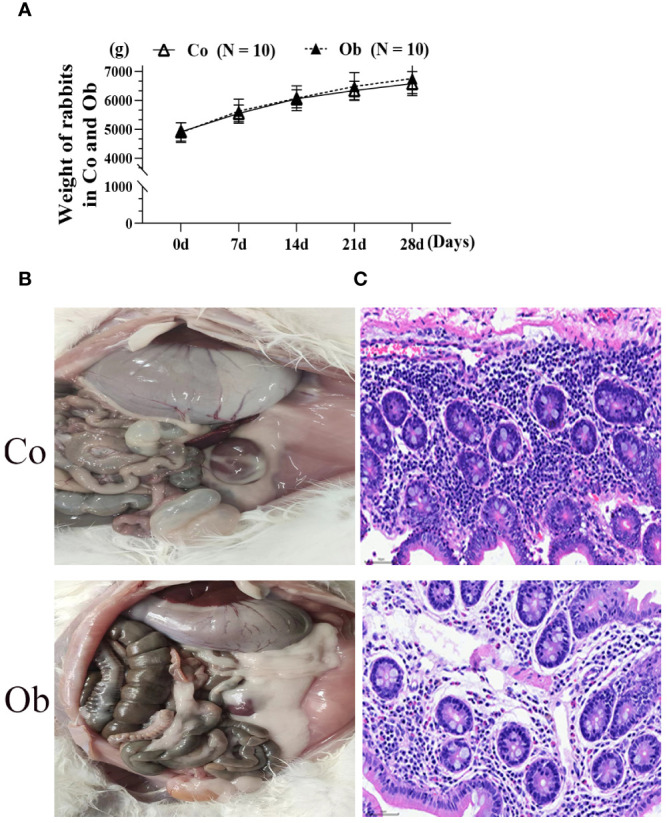
Phenotypic observation and pathological diagnosis of cecum in rabbits under two feeding conditions. **(A, B)** Weight differences and anatomical differences between the two groups of rabbits. **(C)** HE staining at 400×, Bar = 50 μm.

In order to better understand the effects of a high-fat diet on rabbit growth, the body weight, anatomical differences, and cecal pathological diagnosis were used, as shown in **Figure 2**. After four weeks of treatment, the weights of Ob rabbits were higher than the Co rabbits (**Figure 2A**). At the same time, anatomic observation (**Figure 2B**) showed that as compared to the Co group, the perirenal fat of the Ob group rabbits significantly increased. The H&E staining results (**Figure 2C**) showed that the boundaries of the mucosal layer, submucosal layer, muscular layer, and outer membrane of the cecal tissues in the rabbits were clear in both groups. Similarly, the mucosal epithelial cells were arranged neatly and could be seen with a clear striate margin in the free surface. However, as compared to the Co group, there were abundant plasma cells and acidic granulocytes in the subepithelial lamina propria of the cecal mucosa in the Ob rabbits. The results indicated that the high-fat diet not only caused weight gain and visceral fat deposition but also increased the inflammation level of the cecum.

### 3.2 Analysis of the differences in microbial community composition and function between Ob and Co

**Figure 3:**
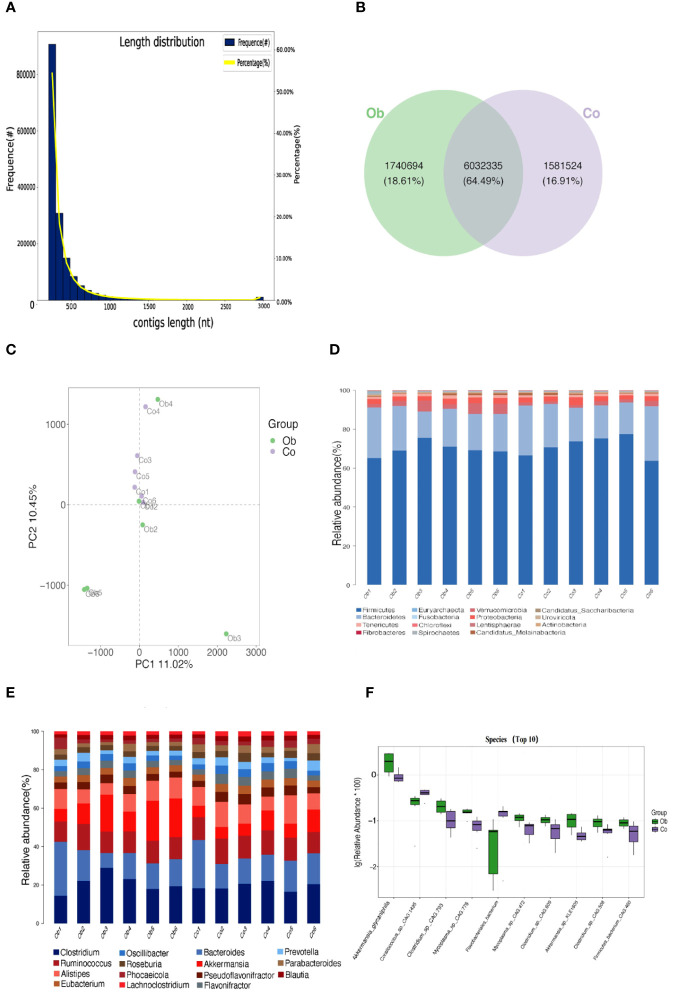
Gene number prediction and microbial differences analysis between the two groups. **(A)** Statistical plot of unigene length distribution. The first vertical axis Frequence (#) represents the number of genes in the gene catalog; the second vertical axis Percentage (%) represents the percentage of genes in the gene catalog; and the horizontal axis represents the length of the genes in the gene catalog. **(B)** Venn diagram (Petal plot) analysis of the differences in the number of genes. **(C)** PCA analysis of the relationship between gene clusters. The horizontal and vertical axes represent the two eigenvalues that maximally reflect the variance. Each point in the figure represents a sample, the same color represents the same group, and similar samples are clustered together. **(D, E)** Significant differences at the phylum and genus levels, respectively. Columns represent samples, and different colors represent different annotation information. A cluster plot of sample similarity. Branch points represent different samples, different colors represent different groups, and the closer the branches, the more similar the samples. **(F)** Box plots showing significantly different species. The horizontal axis is sample grouping. The relative abundance of the corresponding species is shown longitudinally.

Based on the clean read data and genome assembly, ORF prediction was performed on the trimmed contigs sequence (>500bp), and the predicted results are shown in **Figure 3A**. A total of 1,598,336 genes were predicted in the average 592,330,829 bp sequences (The length of contigs was from 201 to 17,907 bp), as shown in **Supplementary Table S1**. The number of genes between the two groups was analyzed, and the results showed that the specific genes in the Ob group were more than those in the Co group (**Figure 3B**). Similarly, the distribution of the gene clusters in a PCA plot revealed different patterns for the Co and Ob rabbits (**Figure 3C**). Furthermore, after comparing to and analyzing with NR database, the differences in the distribution of the top 15 phylum and genus level of samples in each group are shown in **Figures 3D, E**. The results of microbial composition analysis showed that *Firmicutes*, *Bacteroidetes*, *Verrucomicrobia*, *Proteobacteria*, *Tenericutes*, *Candidatus_Melainabacteria*, *Actinobacteria*, *Spirochaetes*, *Euryarchaeota*, and *Fusobacteria* were the most abundance bacterial phyla in the two groups. At the genus level, *Clostridium*, *Bacteroides*, *Ruminococcus*, *Akkermansia*, and *Alistipes* were the most abundant bacterial genera in the two groups. The alpha diversity index, which was determined by calculating the number of reads at the species level in the cecum of the two groups, is shown in **Table 2**. The good coverage index indicated that the coverage rate of the two groups was more than 99%, covering almost the whole gut microbiota in the cecum samples. Chao1, ACE, and Obs indices showed no significant differences between the two groups, while the Shannon and Simpson indices in the Ob group were significantly higher than those in the Co group, indicating relatively higher diversity of gut microbiota in the Ob group than the Co group. The P-values calculated using the Kruskal-Wallis method showed that 10 microbes showed significant differences at the species level in the two groups (**Figure 3F**). These microbes included *Corallococcus_sp._CAG:1435* and *Flavobacteriales bacterium*, which were significantly lower in the Ob group, and *Akkermansia glycaniphila*, *Clostridium_sp._CAG:793*, *Mycoplasma_sp._CAG:776*, *Mycoplasma_sp._CAG:472*, *Clostridium_sp._CAG:609*, *Akkermansia_sp._KLE1605*, *Clostridium_sp._CAG:508*, *Firmicutes_bacterium_CAG:460*, which were significantly higher in the Ob group than the Co group. These diversity analyses indicated that a high-fat diet could increase the diversity of gut microbiota in rabbit cecal tissues.

**Table 2:** Comparative analysis of microbial diversity between the two groups.

Samples	Goods_coverage	Chao1	Shannon	Simpson index	ACE index	Obs
Co	0.9999	18150.8039±223.0546	5.1109±0.0641	0.9569±0.0036	17916.3328±201.8457	16739.3333±209.8766
Ob	0.9999	18215.6193±332.4166	5.1780±0.0123*	0.9625±0.0009^**^	17947.9204±329.2144	16897.3333±325.5156
P-Value	0.104	0.109	0.011	0.009	0.624	0.661

Data was obtained from the cecum contents of two group rabbits. The values were shown as mean ± standard error (Mean ± SE). Values in the same row with different superscripts (“*” and “**”) indicate significant differences between Ob and Co groups (*P* <0.05). All the values were accurate to 0.0001. Obs, observed_species; Chao1, Chao1 richness estimator; Shannon, Shannon index; ACE, Abundance-based Coverage Estimator.

### 3.3 Differences in gut metabolites and their function in the cecum of Ob and Co group rabbits

**Figure 4:**
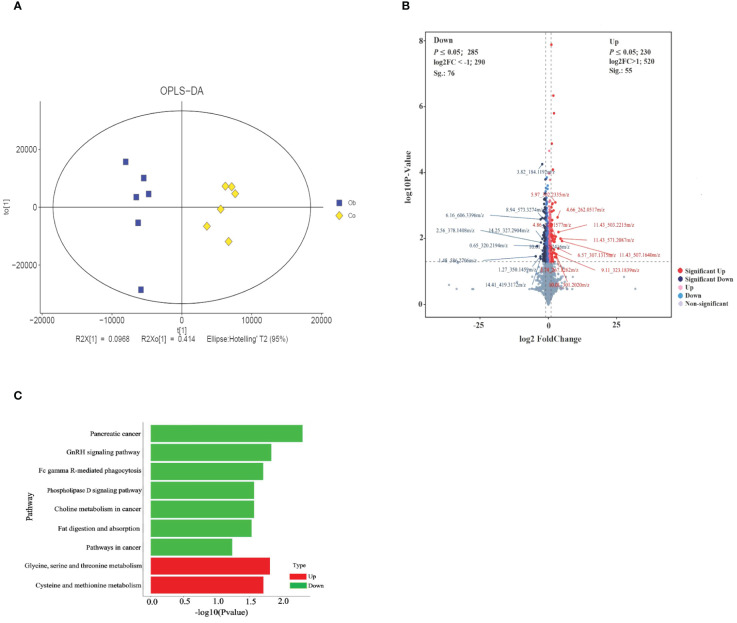
Identification and functional KEGG enrichment analysis of differential metabolites between two groups. **(A)** OPLS-DA for metabolites between groups. **(B)** Volcano plot analysis of differential metabolites. Each point in the figure represents a metabolite, the abscissa is the log2 (FC) value of the comparison between the two groups, the ordinate is the -log10 (P-value) value, and the red point is the significantly upregulated differential metabolites (*P <*0.05, VIP >1, and log2Foldchange>1). Blue dots are significantly downregulated differential metabolites (*P <*0.05, VIP >1, and log2Foldchange<-1). **(C)** Function enrichment analysis of differential metabolites.

The results of OPLS-DA showed that the values of model evaluation parameters were more than O.5 (R2 = 0.704 and Q2 = 0.62), indicating differences in the metabolites between the two groups, which were subsequently analyzed in accordance with the model conditions, as shown in the **Figure 4A**. Based on the threshold values |log2 (FC)| >1 and *P <*0.05 of the metabolites, the top 10 upregulated and downregulated differentially expressed metabolites were obtained (**Figure 4B**; **Table 3**). Then, the functional enrichment analysis of differential metabolites between the two groups showed that the upregulated differential metabolites were mainly enriched in glycine, serine, and threonine metabolism and cysteine and methionine metabolism, while the downregulated differential metabolites were mainly enriched in pancreatic cancer, GnRH signaling pathway, Fc gamma R-mediated phagocytosis, phospholipase D signaling pathway, choline metabolism in cancer, fat digestion and absorption, and cancer pathways (**Figure 4C**). Therefore, these results indicated that the glycine, serine, and threonine metabolism and cysteine and methionine metabolism were important metabolic enrichment pathways in response to hyperlipid metabolism.

**Table 3:** Differential metabolites between the two groups.

ID	Metabolites	log2FoldChange	P-value	VIP	Regulation
10.01_301.2020m/z	Lyngbic acid	2.730768091	0.02948	0.71126	Up
11.43_503.2215m/z	(1Z,4Z)-1,5-bis(4-hydroxyphenyl)-1,4-pentadiene	3.690065211	0.00637	0.97238	Up
11.43_571.2087m/z	RepSox	4.446489515	0.01002	0.7788	Up
4.86_304.1577m/z	ZOLAZEPAM	2.69362292	0.00875	0.93751	Up
11.43_507.1640m/z	Indolocarbazole	4.979854712	0.01186	0.61991	Up
6.57_307.1315m/z	2-(4-Amino-1-isopropyl-1H-pyrazolo[3,4-d]pyrimidin-3-yl)-1H-indol-5-ol	3.597126654	0.01992	0.42084	Up
3.74_267.1262m/z	Indalpine	2.913154151	0.03719	0.64023	Up
9.11_323.1839m/z	1-(4-Hydroxy-3-methoxyphenyl)-3-decanone	2.935464744	0.03011	0.57204	Up
4.66_262.0517m/z	7-Acetamido-9-oxofluorene-4-carboxylic acid	3.365220685	0.00227	0.26844	Up
5.97_302.2335m/z	N-Dodecylsarcosinate	2.562323306	0.0008	0.69331	Up
1.27_350.1459m/z	3-Hydroxyhexanedioylcarnitine	-2.87871226	0.03474	1.18534	down
14.25_327.2904m/z	3-hydroxyicosanoic Acid	-2.20412688	0.01029	1.49526	down
2.56_378.1408m/z	8-methylthiooctyldesulfoglucosinolate	-2.692726398	0.01316	1.74411	down
14.41_419.3172m/z	4-tridecynoic acid	-2.838463639	0.04052	0.46346	down
8.94_573.3274m/z	Phenbenzamine	-2.187088434	0.00238	0.67982	down
0.65_320.2194m/z	N-Lauroyl Proline	-4.098781825	0.01708	0.51397	down
1.48_586.2706m/z	Diethylaminoethyl-dextran	-4.620029535	0.03461	2.75523	down
10.61_467.2416m/z	PA(17:1(9Z)/0:0)	-2.073282412	0.02984	2.59719	down
3.82_184.1192m/z	Bethanechol	-2.250843034	5.50E-05	0.80099	down
6.16_606.3396m/z	PHOOA-PE	-2.832505411	0.00261	0.88887	down

### 3.4 Differential host gene analysis of cecal tissues between the two groups

**Figure 5:**
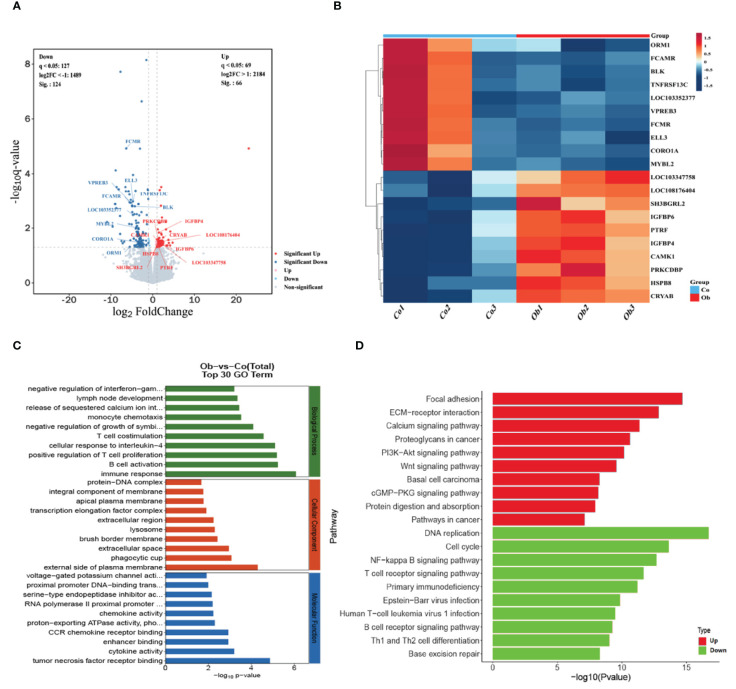
Screening and functional enrichment analysis of differential genes between the two groups. **(A)** Volcano plot analysis of DEGs between the two groups. The gray color represents the non-significant DEGs, and the red and blue colors represent the significant DEGs. The horizontal axis shows the log2Foldchange, and the vertical axis shows the -log10q-value. **(B)** Heat map of differential gene expression in different groups. Blue to orange-red represent the transition from weak to strong gene expression. **(C, D)** The differential genes involved in the top-ranked GO functional enrichment pathway and KEGG pathway.

The transcriptomic sequencing of the two groups of cecal samples showed that the effective data amount of each sample was distributed in 6.86-7.38 G, which contained the Q30 base (distributed in 93.99-94.38%) and average GC content (53.22%). Each sample was aligned to the reference genome (GCF_009806435.1) with an alignment rate of 82.29-88.83%. It was found that the number of coding genes in each sample ranged from 15,554 to 16,056. The main transcriptional sequencing information statistics were shown in the **Supplementary Table S2**. By comparing the expression levels of genes between the two groups, a total of 15,963 differentially expressed genes (DEGs) were found, including 190 significant DEGs (66 upregulated and 124 downregulated), as shown in **Figure 5A**. Then, twenty highly significant differential genes were selected for heat map analysis, as shown in **Figure 5B**. GO enrichment analysis showed the enrichment of these DEGs in the following GO terms: biological processes, including immune response, B cell activation, positive regulation of T cell proliferation, cellular response to interleukin-4, and T cell co-stimulation; cellular components, including external side of plasma membrane, phagocytic cup, extracellular space, brush border membrane, lysosome, extracellular region, and transcription elongation factor complex; molecular functions, including tumor necrosis factor receptor binding, cytokine activity, enhancer binding, CCR chemokine receptor binding, proton-exporting ATPase activity, phosphorylative mechanism, chemokine activity, and RNA polymerase II proximal promoter sequence-specific DNA binding, as shown in **Figure 5C**. KEGG pathway enrichment analysis showed that the upregulated DEGs (*P <*0.05) were mainly involved in the pathways, including focal adhesion, ECM-receptor interaction, calcium signaling pathway, PI3K-Akt signaling pathway, and Wnt signaling pathway, while the downregulated DEGs (*P <*0.05) were mainly enriched in the pathways, including DNA replication, cell cycle, NF-kappa B signaling pathway, and T cell receptor signaling pathway, as shown in **Figure 5D**. Therefore, the results indicated the high-fat diet could cause an increase in metabolic response and a decrease in immune ability in the cecum of rabbits.

### 3.5 Effects of host gene and gut microbiota interaction on gut metabolites in rabbit cecal tissues

**Figure 6:**
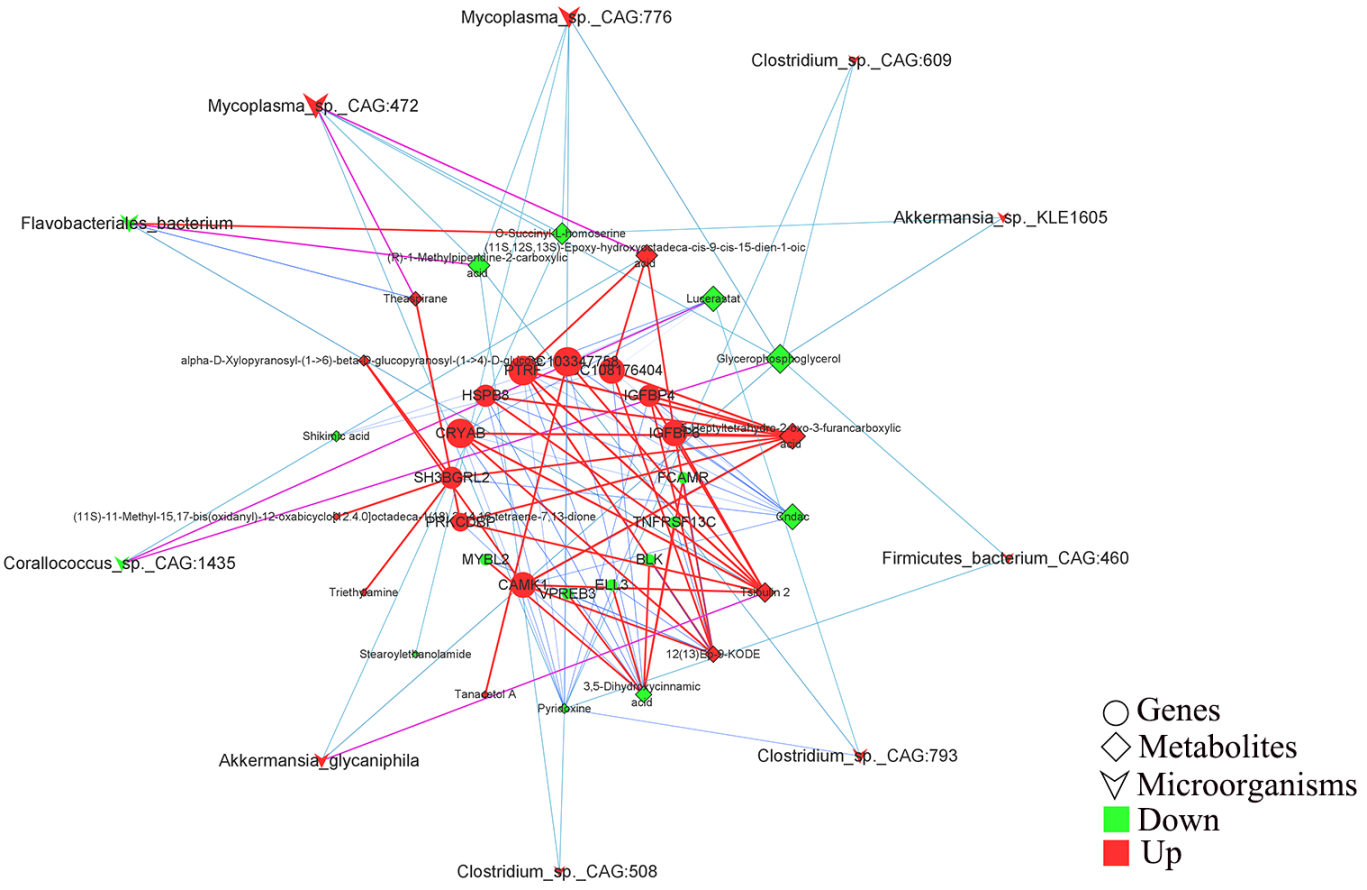
Correlation analysis of the host differential genes, differential metabolites and differential microorganisms for constructing the combine network. The circles, prisms, and swords represent the selected genes, metabolites, and microorganisms, respectively. Red and green represent that the mean expression level of this parameter between Co and Ob is increased and decreased, respectively. The changes of the line from blue to red respectively represents the weak to strong correlation tendency between the two parameter.

Pearson correlation analysis was performed to construct the correlation expression matrix using the top 20 differential genes, metabolites, and microbiota between the two groups (**Supplementary Figures S1A, B**). The correlation analysis between genes and metabolites showed that the genes, including *SH3BGRL2*, *LOC108176404*, *LOC103347758*, *IGFBP4*, *IGFBP6*, *HSPB8*, *PRKCDBP*, *CRYAB*, *PTRF*, and *CAMK1*, were positively correlated with the metabolites, including triethylamine, (11S,12S,13S)-epoxy-hydrox yoctadeca-cis-9-cis-15-dien-1-oic acid, 12 (13)Ep-9-KODE, tsibulin 2, 5-heptyltetrahydro-2-oxo-3-furancarboxylic acid, theaspirane, tanacetol A (11S) -11-methyl-15,17-bis(oxidanyl)-12-oxabicyclo[12.4.0]octadeca-1 (18),2,14,16-tetraene-7,13-dione, and alpha-D-Xylopyranos yl-(1->6)-beta-D-glucopyranosyl-(1->4)-D-glucose) and negatively correlated with the other selected metabolites. Similarly, *ELL3*, *FCAMR*, *MYBL2*, *TNFRSF13C*, *VPREB3*, *BLK*, *ORM1*, *LOC103352377*, *CORO1A*, and *FCMR* genes were positively correlated with glycerophosphoglycerol, shikimic acid, pyridoxine, lucerastat, 3,5-dihydroxycinnamic acid, and cndac and negatively correlated with the other metabolites. Importantly, (R)-methylpiperidine-2-carboxylic acid, 3’-ketolactose, O-succinyl-L-homoserine, stearoylethanolamide, and N-acetyl-L-aspartic acid were negatively correlated with the selected DEGs. The correlation analysis between microorganisms and metabolites showed that *Monoglobus_pectinilyticus*, *Corallococcus_sp._CAG:1435*, *Coprobacter_secundus*, and *Flavobacteriales_bacterium* were negatively correlated with tanacetol A, (11S)-11-methyl-15,17-bis(oxidanyl)-12-oxabicyclo[12.4.0]octadeca-1 (18),2,14,16-Tetraene-7,13-dione, 5-Heptyltetrahydro-2-oxo-3-furancarboxylic acid, triethylamine, (11S,12S,13S)-hydroxyoctadeca-cis-9-cis-15-dien-1-oic acid, 12 (13) Ep-9-KODE, theaspirane, and Tsibulin 2 and positively correlated with the other metabolites. On the contrary, the other differential gut microbiota, including *Eubacterium_sp._CAG:180*, *Akkermansia_SP._BIOM-A60*, *Akkermansia_sp._KLE1605*, *Akkermansia_glycaniphila*, etc., were positively correlated with the differential metabolites, including tanacetol A, (11S)-11-methyl-15,17-bis(oxidanyl)-12-oxabicyclo[12.4.0]octadeca-1 (18),2,14, 16-Tetraene-7,13-dione, 5-Heptyltetrahydro-2-oxo-3-furancarboxylic acid, triethylamine, (11S,12S,13S)-hydroxyoctadeca-cis-9-cis-15-dien-1-oic acid, 12 (13)Ep-9-KODE, theaspirane and tsibulin 2 and negatively correlated with stearoylethanolamide, shikimic acid, cndac, lucerastat, and glycerophosphoglycerol. Furthermore, the correlation network analysis (**Figure 6**) among the DEGs, differential metabolites, and microbiota showed that *HSPB8*, *LOC103347758*, *CRYAB*, *LOC108176404*, *PTRF*, *IGFBP6*, *CAMK1*, *PRKCDBP*, *SH3BGRL2*, and *IGFBP4* genes were highly correlated with the gut microbiota, including *Corallococcus_sp._CAG:1435*, *Mycoplasma_sp._CAG:472*, *Flavobacteriales_bacterium*, *Clostridium_sp._CAG:793*, and *Akkermansia_glycaniphila*, through the metabolites, including (11S,12S,13S)-Epoxy-hydroxyoctadeca-cis-9-cis-15-dien-1-oic acid, lucerastat, 5-Heptyltetrahydro-2-oxo-3-furancarboxylic acid, Cndac, and Tsibulin 2. The details of this network diagram are shown in **Table 4**. Therefore, these results indicated that the interactions between gut microbiota and host genes played an important role in regulating lipid degradation in the cecum of rabbits.

**Table 4:** Information on the expression parameters of key genes, microorganisms and metabolites in the correlation network graph.

Name	Type	Degree	Edge Count	Neighborhood Connectivity	Number Of Undirected Edges	Radiality	Topological Coefficient	Regulation
Clostridium_sp._CAG:793	Microbe	3	3	8.3333	3	0.8688	0.4889	up
Mycoplasma_sp._CAG:472	Microbe	6	6	6.5000	6	0.8887	0.3056	up
Flavobacteriales_bacterium	Microbe	4	4	6.0000	4	0.8721	0.3333	down
Corallococcus_sp._CAG:1435	Microbe	3	3	6.0000	3	0.8555	0.4167	down
Akkermansia_glycaniphila	Microbe	3	3	7.6667	3	0.8721	0.4167	up
CAMK1	Gene	6	6	9.5000	6	0.9120	0.3696	up
PRKCDBP	Gene	4	4	8.7500	4	0.8854	0.4306	up
SH3BGRL2	Gene	5	5	4.4000	5	0.8455	0.3778	up
CRYAB	Gene	7	7	10.4286	7	0.9186	0.3929	up
HSPB8	Gene	5	5	11.0000	5	0.9086	0.4348	up
PTRF	Gene	8	8	9.7500	8	0.9219	0.3646	up
LOC103347758	Gene	7	7	7.8571	7	0.9186	0.2857	up
LOC108176404	Gene	9	9	8.8889	9	0.9252	0.3287	up
IGFBP4	Gene	5	5	11.0000	5	0.9086	0.4348	up
IGFBP6	Gene	6	6	10.0000	6	0.9153	0.3750	up
Pyridoxine	Metabolite	14	14	5.2143	14	0.9252	0.2810	down
Lucerastat	Metabolite	6	6	6.1667	6	0.8854	0.4697	down
(11S,12S,13S)-Epoxy-hydroxyoctadeca-cis-9-cis-15-dien-1-oic acid	Metabolite	5	5	6.4000	5	0.8854	0.4500	up
Tsibulin 2	Metabolite	11	11	5.8182	11	0.9120	0.3442	up
Cndac	Metabolite	9	9	6.4444	9	0.8953	0.4537	down
5-Heptyltetrahydro-2-oxo-3-furancarboxylic acid	Metabolite	9	9	6.1111	9	0.8953	0.4259	up

## 4 Discussion

The high-calorie eating habit is an important factor in obesity (Castro-Barquero et al., 2020; Hemmer et al., 2021). A previous study found that a high-fat diet could decrease the villi length of the small intestine and colon in 12-month-old female mice, resulting in impaired epithelial barrier function (Xie et al., 2020). Similarly, the high-fat diet can also increase the pro-inflammatory gut microbiota, directly affecting the pH value in the intestinal environment, promoting intestinal permeability, and enhancing the lipopolysaccharide levels in the process of circulating metabolism (Beam et al., 2021). The high-fat diet can cause an imbalance of intestinal immune regulation, which results in the infiltration of toxic microbial metabolites, causing systemic inflammation (Malesza et al., 2021). Clinical studies have also shown that plasma cells and acidic granulocytes, as inherent immune cells of the human body, participate in the inflammatory process of body tissues and actively respond to regulating the immune metabolism caused by lipid metabolism (D'Souza and Bhattacharya, 2019; Li et al., 2021). Eosinophils can secrete a large number of anti-inflammatory factors, balance the immune homeostasis of the intestinal mucosal layer, and promote the formation of intestinal mucosal villi (Li et al., 2023). The current study also showed that the short-term high-fat diet increased body weight, kidney fat, and intestinal fat in rabbits. The pathological diagnosis showed that there were abundant plasma cells and acidic granulocytes in the mucous membrane of the cecum intestinal epithelium, suggesting changes in the homeostasis and immune environment in rabbit cecum. Previous studies indicated that the diversity of gut microbiota in obese patients was lower than that the normal healthy people, which affected the production of metabolites and expression level of host intestinal genes (Amabebe et al., 2020; Zhou et al., 2020). Particularly, Bacteroidetes and Firmicutes in the gut microbiota of obese people can promote the absorption of excess energy, and their relative abundances are closely related to intestinal metabolism (Turnbaugh et al., 2006). The Firmicutes to Bacteroidetes (F/B) ratio is considered a marker of obesity (Grigor'eva, 2020). In addition, *Blautia*, *Romboutsia*, *Ruminococcus2*, *Clostridium sensu stricto*, and *Dorea genera* were positively correlated with the serum indices of obese people, such as low-density lipoprotein, triglyceride, and total cholesterol, while the genera *Bacteroides*, *Roseburia*, *Butyricicoccus*, *Alistipes*, *Parasutterella*, *Parabacteroides*, and *Clostridium IV* were negatively correlated with these serum indices (Zeng et al., 2019). Other studies have shown that *Akkermansia muciniphila*, which colonizes the intestinal mucosal layer in humans and rodents, plays a vital role in maintaining the integrity of the intestinal mucosal layer and intestinal and triglyceride concentration in obese individuals (Parks et al., 2013; Reunanen et al., 2015). The current study found an increase in the relative abundances of Clostridium_sp. (*Clostridium_sp._CAG:793/609/508*), *Akkermansia_sp._KLE1605*, and *Firmicutes_bacterium_CAG:460* in the Ob rabbits. Therefore, it was speculated that these changes in gut microbiota induced by a high-fat diet might indirectly participate in lipid metabolism. Studies have found that a high-fat diet could disrupt the interaction between the local intestinal mucosal immune system and the gut microbiota, resulting in the imbalance of the composition of gut microbiota, especially increasing the number of *Gram-negative bacteria*. *Gram-negative bacteria* can produce lipopolysaccharides that interact with the CD14/TLR4 complex of intestinal epithelial cells, activating the innate immune system (Li et al., 2022). Furthermore, this activation causes persistent low-level inflammation in the body, leading to the destruction of the mucosal layer and increasing the intestinal epithelial cells permeability. This promotes the entry of intestinal microbial metabolites into the bloodstream, resulting in a vicious cycle of inflammation and microbial imbalance (Ornelas et al., 2022). In the current study, the differential analysis of gut microbiota indicated that the relative abundance of Mycoplasma_sp. (*Mycoplasma_sp._CAG:776/472*) increased in the cecum of obese rabbits. Therefore, based on the cecum histopathological diagnosis, it was speculated that these bacteria might cause the inherent immune activation of rabbit cecum epithelial cells. A previous study found that the *Corallococcus* sp. strain was correlated with glucose metabolism in dietary starch and played an important regulatory role in the degradation of crude fiber in the body (Ye et al., 2022). The cecum of rabbits is an important place for crude fiber digestion. The increase in lipids in a high-fat diet increases the relative abundance of lipid metabolism-related microbiota. Therefore, it was suggested that the decline in the relative abundance of *Corallococcus_sp._CAG:1435* and *Flavobacteriales_bacterium* was correlated with the degradation and digestion of crude fiber in the cecum.

The fermentation of nutrients and secondary metabolites by gut microbiota directly affects immunity, metabolic homeostasis, and lipid accumulation in intestinal epithelial cells. Previous studies have shown that the high-fat diet-induced obese mice exhibit altered gut microbiota and metabolic pathways, especially the changes in the relative abundance of Firmicutes, which can influence the production of metabolites, such as serum inositol, tyrosine, and glycine, and upregulate the metabolic pathways, including glycine, serine and threonine metabolism and cysteine and methionine metabolism (Jo et al., 2021). Similarly, it was also found that a high-content DHA diet could alleviate metabolic disorders in obese mice through key metabolic pathways of gut microbiota, including glycerolipid metabolism and glyoxylate and dicarboxylate metabolism (Zhang et al., 2022). In addition, the abnormal amino acid metabolism might be an important factor causing inflammation in the body. The serum levels of histidine and arginine were negatively correlated with inflammation and oxidative stress in obese women as compared to non-obese women (Niu et al., 2012). The plasma level of glycine was lower in obese patients and positively correlated with insulin sensitivity (Adeva-Andany et al., 2018). In this study, glycine, serine, and threonine metabolism and cysteine and methionine metabolism pathways were found to be the main pathways enriched by metabolites in the cecum of obese rabbits. This suggested that the metabolism relationship between the bacteria and the host was more inclined to strengthen the process of lipid metabolism so as to maintain the normal metabolic function in the cecum.

Adipose tissue is an endocrine organ that can secrete anti-inflammatory factors (Adiponectin) and pro-inflammatory factors in different parts, which can cause inflammatory bowel disease (Johnson and Loftus, 2021). The difference in gut microbiota and host genes induced by high lipids is probably inclined to the pathological process of chronic inflammatory response and metabolic disorders. A study showed that PI3K)/Akt/NF-κB signaling pathways were an important pathway in regulating cell proliferation and apoptosis (Huang et al., 2018). In obesity, fatty acid overload in adipose tissue can cause ectopic lipid deposition. The PI3K/AKT pathway can be activated to mediate *SREBP* regulation of fatty acid synthesis and FOXO1 signal pathway for fatty triglyceride lipase (ATGL) regulation of lipolysis, increasing glucose utilization and body fat deposition and reducing insulin resistance caused by obesity (Zhang et al., 2018; Savova et al., 2023). Moreover, the NF-κB signaling pathway can be activated by pro-inflammatory factors, apoptotic mediators, metabolic stress, and chemical agents. The activation NF-κB signaling pathway is associated with the PI3K/AKT signal pathway in insulin resistance and pancreatic beta cell dysfunction in metabolic syndrome (Malle et al., 2015; Sabir et al., 2019; Capece et al., 2022). Similarly, the mechanism of insulin resistance stems from the interaction between gut microbiota and host genes. Previous studies reported that the lipid diet-induced gut microbiota, including *Parabacteroides distasonis*, *Bacteroides* spp., and *Lactobacillus* spp., could reduce the *NF-κB* gene expression level as well as the levels of pro-inflammatory Th17 cells in the gut tissues (Ang et al., 2020; Brīvība et al., 2023). The current study found that the cecal host genes involved in the calcium signaling pathway and PI3K-Akt signaling pathway were upregulated, while those involved in the NF-κB signaling pathway and T cell receptor signaling pathway were downregulated. This suggested that the microbial metabolites might play a regulatory role in the inflammatory signaling pathway.

Insulin-like growth factors (IGFs) and IGF-binding proteins (IGFBPs) play an important role in the development of obesity. It was found that *IGFBP4*, as a member of the IGFBP family that regulates the biological activity of IGFs, could induce adipose tissue expansion and enhance lipid deposition in adipose tissue around the groin and gonads in mice (Maridas et al., 2017). In a study of obese children, the serum expression level of *IGFBP4* was higher than that of *IGFBP6*, which was positively correlated with the indices of apelin, cholecystockinin, glucagon-like peptide-1, and leptin receptors (Czogała et al., 2021). A study of gut microbiota showed that *Akkermansia glycaniphila* exhibited a glyco-based hydrolase gene, which could cleave specific glycan bonds in the obese human gastrointestinal tract (Glover et al., 2022). The current study found that the expression levels of *IGFBP4* and *IGFBP6* were upregulated in the cecum tissues of obese rabbits. This suggested that *Clostridium_sp._CAG:793* and *Akkermansia glycaniphila* might be involved in the metabolic pathway of *IGFBP6* during the regulation of cecum metabolism in obese rabbits. A previous study found that CaMK1, a class of protein kinases, could regulate protein phosphorylation by binding to Ca (2+)/calmodulin, and the polymorphism in the calmodulin-dependent kinase isomer D (CaMK1D) gene was associated with the development of diabetes as well as improved insulin sensitivity and glycemic control in obese mouse model (Fromont et al., 2020). The polymorphisms in the *PRKCDBP* gene can be used as a transcriptional target for *TNF-α* to cause intestinal inflammatory diseases (Kim et al., 2015). In the current study, the interaction between the upregulation of *CaMK1* and *PRKCDBP* genes and these microbes (*Mycoplasma_sp._CAG:472*, *Clostridium_sp._CAG:793*, and *Akkermansia glycaniphila*) in the cecum were also highly correlated in the correlation network, suggesting a key regulatory function of lipid metabolism and the immune response in the mucosal layer of the cecum. Different dietary habits can affect the changes of intestinal flora. Previous study have shown that high-fat diet can increase the proportion of intestinal microbes (*Firmicutes*, *Prevotella*, and *Methanobrevibacter* to enhance fat metabolism and reduce the proportion of beneficial microorganisms, such as *Bacteroides*. *Lactobacillus*, and *Akkermansia* (Amabebe et al., 2020). However, gastrointestinal microbes of different intestinal segments in cattle, sheep, and panda have existed the differences for digesting crude fiber diet, such as *Bacteroidetes*, *Firmicutes* and *Proteobacteria*, *Streptococcus*, *Clostridium*, *Escherichia* (Paradiso et al., 2021; Deng et al., 2023; Long et al., 2024). In this study, three key microbial species in cecum (*Mycoplasma_sp._CAG:472*, *Clostridium_sp._CAG:793*, and *Akkermansia glycaniphila*) were inconsistent with the above research results. Its reasons may be related to differences in gut microbes among species, the selection of intestinal samples, and food-borne microbial characteristics. Besides, this study also found that two rabbit-specific genes (*LOC103347758* and *LOC108176404*) had high weight coefficients in the correlation network, which might play an important role in regulating the metabolism of rabbit gut microbiota. However, there were still several limitations in this study, which failed to conduct in-depth analysis of intestinal flora differences, cecal metabolite formation mechanism, and functional verification using gene knockout technology for comprehensively analyze the regulatory effects of rabbit intestinal flora and gene interaction on lipid metabolism, so as to provide experimental basis for the treatment of human obesity in the future.

## Data availability statement

The data presented in the study are deposited in the NCBI repository, accession number PRJNA1119243 and PRJNA1119171.

## Ethics statement

The animal study was approved by the ethical standards of the Animal experimental ethical inspection form of Guizhou Medical University. The study was conducted in accordance with the local legislation and institutional requirements. This study was approved by and conducted in strict accordance with the ethical standards of the Animal experimental ethical inspection form of Guizhou Medical University, Guizhou, China (No: 2201379).

## Author contributions

YL: Writing – review & editing, Writing – original draft, Project administration, Methodology, Conceptualization. XQ: Writing – review & editing, Methodology, Funding acquisition, Conceptualization. QW: Writing – review & editing, Methodology, Funding acquisition, Conceptualization. YH: Writing – review & editing, Methodology, Funding acquisition, Conceptualization. ZL: Writing – review & editing, Software. XC: Writing – review & editing, Software. LW: Writing – review & editing, Project administration, Methodology, Conceptualization.

## Supplementary material

Supplementary Figure 1 Correlation analysis of host differential genes, differential metabolites and differential microorganisms. **(A, B)** Red and blue dots represents the strong and weak relationships between the two corresponding parameters on the axis, respectively.
