# Supplementary figures and images for: Comprehensive analysis of key host gene-microbe networks in the cecum tissues of the obese rabbits induced by a high-fat diet
Source: Front Cell Infect Microbiol. 2024 Jun 14;14:1407051. doi: 10.3389/fcimb.2024.1407051 (PMC11211605; doi:10.3389/fcimb.2024.1407051)

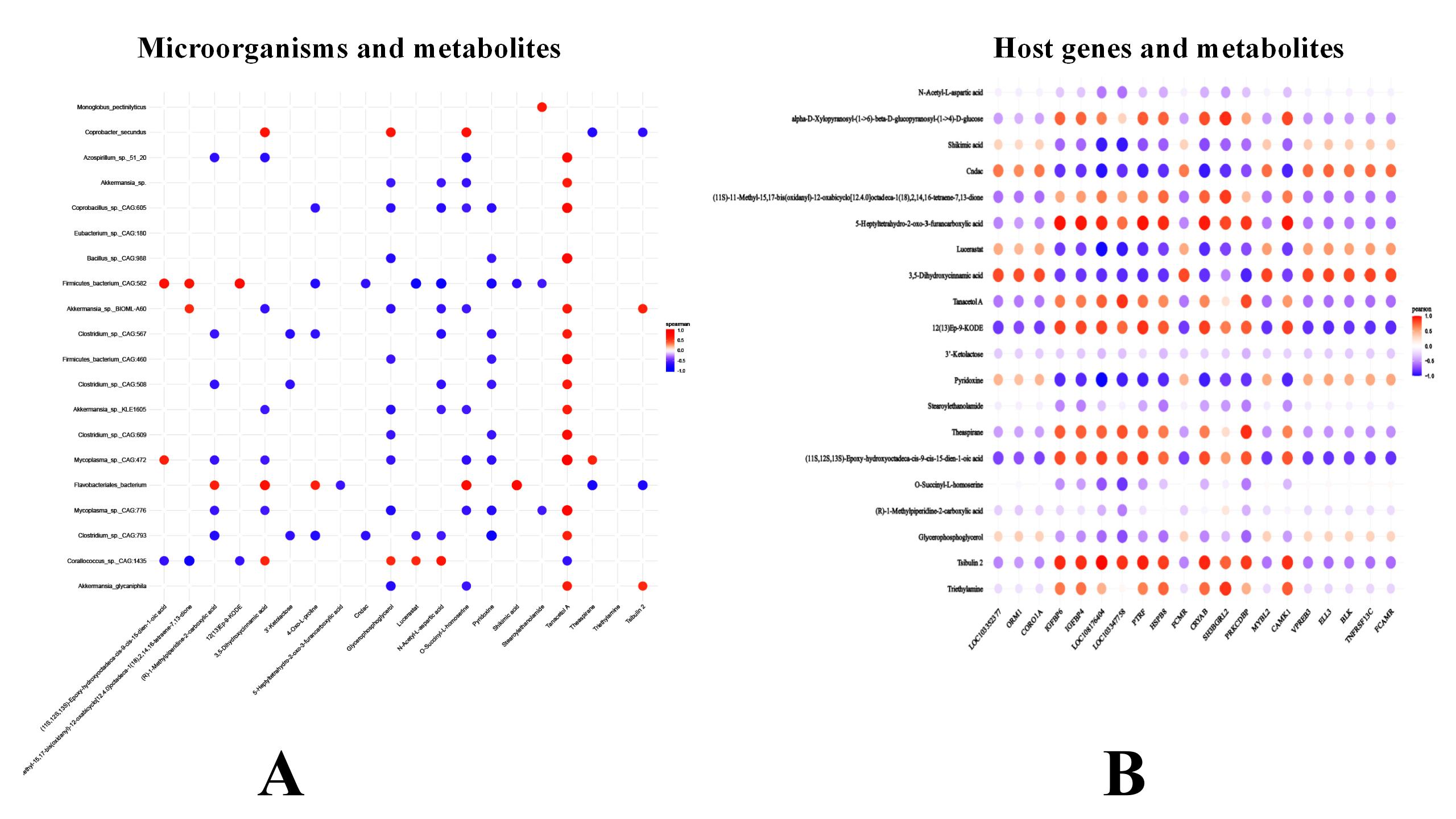

Supplement: Supplementary Figure 1 — Correlation analysis of host differential genes, differential metabolites and differential microorganisms. (A, B) Red and blue dots represents the strong and weak relationships between the two corresponding parameters on the axis, respectively. [file Image_2.jpeg]
